# Low UGP2 Expression Is Associated with Tumour Progression and Predicts Poor Prognosis in Hepatocellular Carcinoma

**DOI:** 10.1155/2020/3231273

**Published:** 2020-07-11

**Authors:** Qiuyue Hu, Shen Shen, Jianhao Li, Liwen Liu, Xin Liu, Yingying Zhang, Yongjian Zhou, Weiwei Zhu, Yan Yu, Guangying Cui

**Affiliations:** ^1^Department of Infectious Diseases, The First Affiliated Hospital of Zhengzhou University, Zhengzhou 450052, China; ^2^Gene Hospital of Henan Province, Precision Medicine Center, The First Affiliated Hospital of Zhengzhou University, Zhengzhou 450052, China

## Abstract

Hepatocellular carcinoma (HCC) is a malignant tumour associated with a high mortality rate and poor prognosis worldwide. Uridine diphosphate-glucose pyrophosphorylase 2 (UGP2), a key enzyme in glycogen biosynthesis, has been reported to be associated with the occurrence and development of various cancer types. However, its diagnostic value and prognostic value in HCC remain unclear. The present study observed that UGP2 expression was significantly downregulated at both the mRNA and protein levels in HCC tissues. Receiver operating characteristic (ROC) curve analysis revealed that UGP2 may be an indicator for the diagnosis of HCC. In addition, Kaplan-Meier and Cox regression multivariate analyses indicated that UGP2 is an independent prognostic factor of overall survival (OS) in patients with HCC. Furthermore, gene set enrichment analysis (GSEA) suggested that gene sets negatively correlated with the survival of HCC patients were enriched in the group with low UGP2 expression levels. More importantly, a significant correlation was identified between low UGP2 expression and fatty acid metabolism. In summary, the present study demonstrates that UGP2 may contribute to the progression of HCC, indicating a potential therapeutic target for HCC patients.

## 1. Introduction

Hepatocellular carcinoma (HCC) is one of the most common malignancies and ranks as the second leading cause of cancer-associated mortality worldwide [1–3]. Furthermore, HCC patients have a poor prognosis, with a 5-year survival rate of 18% [4–6]. Early diagnosis is essential to improve the prognosis of patients [7, 8]. Therefore, it is essential to discover novel biological markers for the detection of early HCC and for the prediction of a subset of patients with a high risk of recurrence and/or poor survival outcomes.

Uridine diphosphate-glucose pyrophosphorylase 2 (UGP2), an enzyme that consists of 508 amino acid residues with a relative molecular weight of 56,940 Da, plays a vital role in glycogen biosynthesis. UGP2 catalyses the reaction of glucose-1-phosphate uridylyltransferase and glucose-1-P to produce UDP-glucose, which acts as a glucose donor to participate in the anabolism of sucrose, glycolipids, cellulose, and glycoproteins [9–11]. UGP2 has been reported to be highly expressed in skeletal muscles and the liver and is involved in the process of glycogenesis in muscles and the liver. Previously, several studies have reported the relationship between UGP2 and the occurrence and development of several tumours, including pancreatic ductal carcinoma [12], gallbladder cancer [13], colorectal cancer [14], acute myeloid leukaemia [15], and glioma [16]. Additionally, Tan et al. [17] reported that low UGP2 expression can differentiate between metastatic relapse (MR) HCC patients and nonrelapse (NR) HCC patients. However, the expression of UGP2 and its diagnostic and prognostic value have not been reported in HCC.

The present study identified that UGP2 mRNA and protein expression levels were downregulated in HCC tissues. Additionally, receiver operating characteristic (ROC) curve analyses of UGP2 suggested that UGP2 may be an indicator for the diagnosis of HCC. In addition, Kaplan-Meier and Cox regression multivariate analyses indicated that UGP2 expression is an independent prognostic factor of overall survival (OS) in HCC patients. Furthermore, gene set enrichment analysis (GSEA) revealed that gene sets negatively correlated with the survival of HCC patients were enriched in the group with low UGP2 expression levels. Taken together, these results suggest that the downregulation of UGP2 expression is significantly associated with the progression and poor prognosis of HCC, indicating that UGP2 may provide an approach for early diagnosis and predict prognosis.

## 2. Materials and Methods

### 2.1. The Cancer Genome Atlas (TCGA)/Gene Expression Omnibus (GEO) Dataset Acquisition and Processing

HCC microarray datasets were downloaded from the GEO database (https://www.ncbi.nlm.nih.gov/geo/) for gene expression analysis. A total of 373 HCC patients were obtained from the open access tiers of the TCGA database (https://tcga-data.nci.nih.gov/tcga/), which are referred to as the TCGA cohort in the present study. Among these patients, 318 were included after excluding those with missing UGP2 mRNA expression data and clinical information. The remaining 318 patients were used for gene expression and survival analyses.

### 2.2. Tissue Microarray (TMA) Construction

A pancancer TMA was constructed using the sample library from the First Affiliated Hospital of Zhengzhou University (Zhengzhou, China) to collect ten types of cancer tissues, namely, lung cancer, renal cell carcinoma, oesophageal cancer, thyroid cancer, stomach cancer, rectal cancer, breast cancer, cervical cancer, liver cancer and colon cancer, and paracancerous tissues (approximately 20 pairs of each type of tissue). The TMA procedure was performed as described previously [18].

An HCC follow-up cohort (referred to as the ZZU HCC cohort) containing 396 HCC tissues and paired adjacent normal tissues was acquired from the First Affiliated Hospital of Zhengzhou University between 2011 and 2015. These samples were collected from 335 patients with complete clinical information and UGP2 expression data. The patients consisted of 262 males and 73 females, with a median age of 53 (range, 17-76) years. The clinicopathological characteristics of the patients are summarized in Table 1. The present study was approved by the Research Ethics Committees of the First Affiliated Hospital of Zhengzhou University, and each patient signed an informed consent form.

### 2.3. Immunohistochemical Analysis

The TMA was baked at 62°C, dewaxed with xylene, and hydrated with gradient concentrations of alcohol. After hydration, antigen retrieval was conducted, followed by blocking for 1 h at room temperature. After 30 min of blocking with 10% bovine serum albumin, sections were incubated with a UGP2 primary antibody (1 : 100; ProteinTech Group, Inc.) at 4°C overnight. The following day, the sections were incubated at room temperature for 30 min with the corresponding secondary antibody labelled with peroxidase and washed three times with PBS. Finally, a colour reaction was performed with SignalStain® DAB (Cell Signalling Technology, Inc.), and nuclear counterstaining was performed with haematoxylin QS (Vector Laboratories, Ltd.). Two pathological experts used the double-blind method to evaluate immunostaining. Additionally, a scoring system for UGP2 expression was established. According to the different intensities of UGP2 staining, the expression level of UGP2 was divided into five categories. For the statistical analysis, scores of 1+, 2+, and 3+ were classified as low UGP2 expression levels, while scores of 4+ and 5+ were classified as high UGP2 expression levels.

### 2.4. GSEA and Scatter Plot Analysis

GSEA was used to investigate the potential function of UGP2 in HCC patients. If most members of a gene set were positively correlated with the expression of UGP2, the set was defined as associated with UGP2. *P* < 0.05 was selected as the significance threshold. Scatter plot analysis was used to assess the association between UGP2 and genes involved in the fatty acid metabolism pathway.

### 2.5. Statistical Analysis

SPSS software (version 24.0; IBM Corp.) was used for statistical analysis. The experimental data are expressed as the mean ± standard deviation. Analysis of variance or Student's *t*-test was used for the measurement data analysis. A rank-sum test or a *χ*^2^ test was used for the counting data. Grade correlation analysis was used in the correlation analysis. Kyoto Encyclopedia of Genes and Genomes (KEGG) pathway enrichment analysis was used to detect the potential molecular mechanism of UGP2 in HCC. The log-rank method was used in the survival analysis. A Cox regression multivariate model was used to analyse the prognostic factors. *P* < 0.05 was considered to indicate a statistically significant difference.

## 3. Results

### 3.1. UGP2 mRNA Levels and Protein Expression Are Frequently Downregulated in Cancer

**Figure 1:**
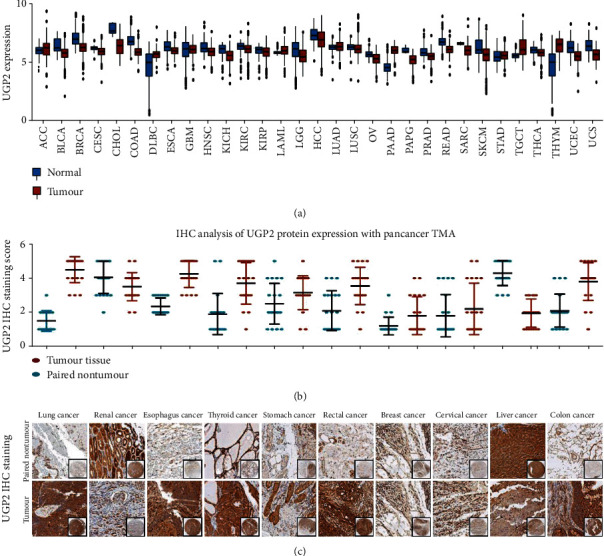
UGP2 expression is frequently dysregulated in cancer. (a) UGP2 mRNA expression level from the TCGA dataset compared with normal tissues. (b) UGP2 protein expression in pancancer tissues and paired nontumour tissues. (c) Representative UGP2 histological scoring in pancancer tissues and paired nontumour tissues. UGP2: uridine diphosphate-glucose pyrophosphorylase 2; TCGA: The Cancer Genome Atlas.

To detect the expression of UGP2 in tumours, UGP2 mRNA expression was investigated in different cancer types using TCGA datasets. The results showed that compared with normal tissues, UGP2 mRNA expression was significantly downregulated in most tumour types, including HCC (*P* < 0.001; Figure 1(a)). Considering that the process from mRNA to protein involves a whole set of expression regulatory mechanisms, such as translational regulation and posttranslational regulation, the protein expression of UGP2 was further determined in a pancancer TMA. Consistent with the UGP2 mRNA expression observed in the TCGA dataset analysis, the pancancer TMA analysis also indicated that the protein expression level of UGP2 in most tumour tissues, including HCC, was lower than that in adjacent nontumour tissues (*P* < 0.001; Figures 1(b) and 1(c)).

### 3.2. UGP2 mRNA and Protein Expression Levels Are Significantly Downregulated in HCC

**Figure 2:**
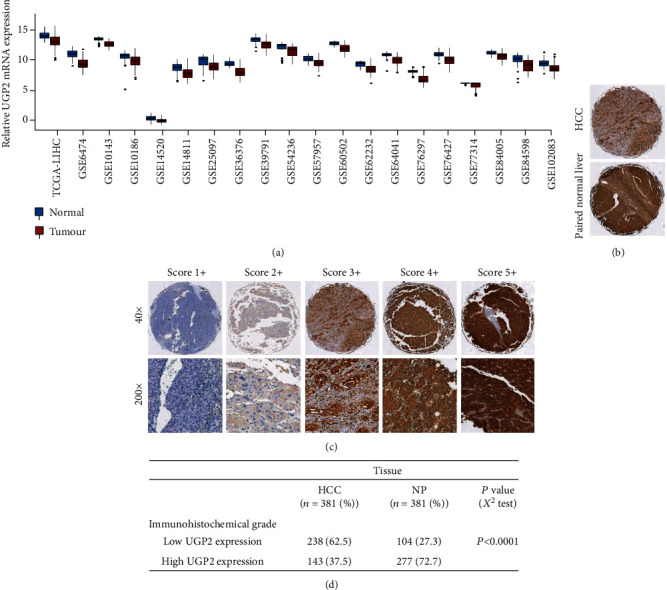
UGP2 mRNA levels and protein expression are significantly downregulated in HCC. (a) The UGP2 expression level was significantly lower in HCC tissues than in adjacent nontumour tissues in the TCGA cohort and GEO datasets. (b) A representative IHC image of UGP2 expression in HCC and normal tissues. (c) Representative images of UGP2 staining in HCC tissues. (d) The UGP2 expression level in HCC tissues was lower than that in paired nontumour tissues in the ZZU HCC cohort. UGP2: uridine diphosphate-glucose pyrophosphorylase 2; TCGA: The Cancer Genome Atlas; HCC: hepatocellular carcinoma; GEO: Gene Expression Omnibus; IHC: immunohistochemistry.

To further confirm the expression status of UGP2 in HCC, TCGA datasets and GEO data were analysed. The results demonstrated that UGP2 expression in HCC tissues was significantly lower than that in matched adjacent nontumour tissues (*P* < 0.001; Figure 2(a)). Considering the regulatory levels of gene expression, 396 pairs of HCC samples (the ZZU HCC cohort) were analysed by immunohistochemistry (IHC). The method of UGP2 staining classification was performed as described previously (Figures 2(b) and 2(c)). The UGP2 expression level in HCC tissues was lower than that in matched adjacent nontumour tissues (62.5 vs. 27.3%; *P* < 0.0001; Figure 2(d)). Collectively, these results indicate that UGP2 expression is significantly downregulated in HCC tissues.

### 3.3. UGP2 May Have Potential Diagnostic Value for HCC

**Figure 3:**
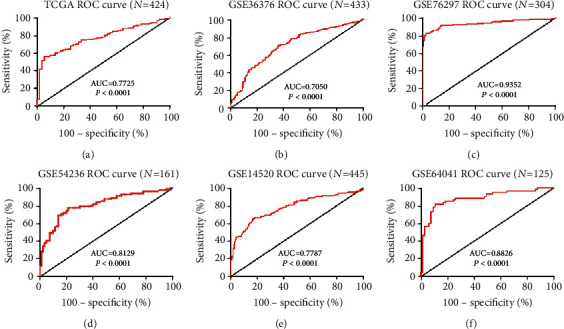
ROC curve analysis of UGP2 expression in HCC. (a–f) ROC curve analysis of UGP2 expression in HCC from the TCGA, GSE36376, GSE76297, GSE54236, GSE14520, and GSE64041 datasets. UGP2: uridine diphosphate-glucose pyrophosphorylase 2; TCGA: The Cancer Genome Atlas; HCC: hepatocellular carcinoma; ROC: receiver operating characteristic.

To analyse the diagnostic accuracy of the UGP2 mRNA expression level for HCC diagnosis, ROC curve analysis was used. In a comparison between tumour tissues and matched adjacent nontumour tissues in the TCGA cohort, the area under the curve (AUC) was 0.7725 (95% confidence interval (CI), 0.717-0.828; *P* < 0.0001; Figure 3(a)). The sensitivity, specificity, positive predictive value (PPV), and negative predictive value (NPV) of UGP2 as a biomarker for the diagnosis of HCC were 56.3%, 94.1%, 98.6%, and 62.5%, respectively. Consistent with the abovementioned results, it was further verified that UGP2 mRNA expression specifically distinguished the HCC tissue group from the matched adjacent nontumour tissue group in five independent HCC cohorts from the GEO database: GSE36376 (AUC, 0.7050; *P* < 0.0001; sensitivity, 70.0%; specificity, 64.3%; PPV, 70.9%; and NPV, 63.3%; Figure 3(b)), GSE76297 (AUC, 0.9352; *P* < 0.0001; sensitivity, 82.4%; specificity, 97.4%; PPV, 96.6%; and NPV, 84.5%; Figure 3(c)), GSE54236 (AUC, 0.8129; *P* < 0.0001; sensitivity, 77.8%; specificity, 78.8%; PPV, 78.5%; and NPV, 76.8%; Figure 3(d)), GSE14520 (AUC, 0.7787; *P* < 0.0001; sensitivity, 65.3%; specificity, 82.7%; PPV, 81.7%; and NPV, 70.0%; Figure 3(e)), and GSE64041 (AUC, 0.8826; *P* < 0.0001; sensitivity, 81.7%; specificity, 89.2%; PPV, 87.7%; and NPV, 85.3%; Figure 3(f)). Thus, these data demonstrated that UGP2 may have potential diagnostic value for HCC.

### 3.4. Downregulated UGP2 Expression Predicts a Poor Prognosis in HCC Patients

**Figure 4:**
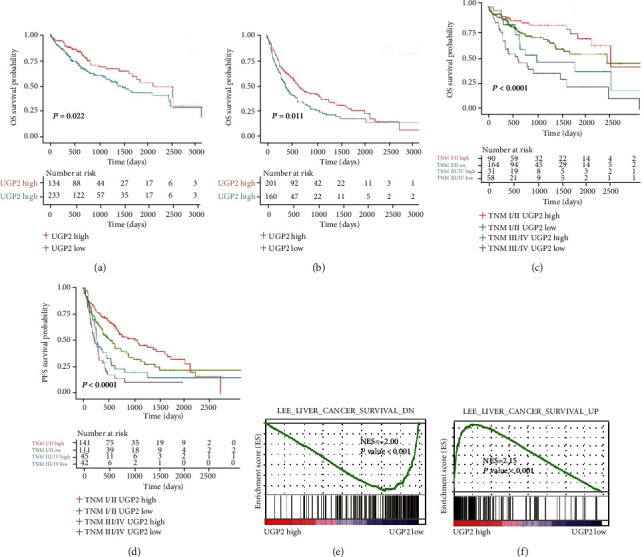
Downregulated UGP2 expression predicts a poor prognosis in HCC patients in the TCGA cohort. (a, b) Kaplan-Meier survival curves showing the correlation between UGP2 expression levels and the OS/PFS of HCC patients in the TCGA cohort. (c, d) Kaplan-Meier analysis showed that OS/PFS was shorter in HCC patients in the TCGA cohort with low UGP2 expression levels regardless of the TNM stage. (e, f) GSEA of the relationship between low UGP2 expression levels and the survival of HCC patients in the TCGA cohort. UGP2: uridine diphosphate-glucose pyrophosphorylase 2; TCGA: The Cancer Genome Atlas; HCC: hepatocellular carcinoma; OS: overall survival; PFS: progression-free survival; HCC: hepatocellular carcinoma.

**Figure 5:**
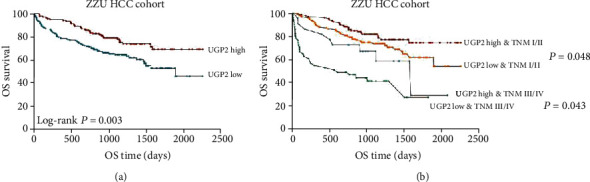
Downregulated UGP2 expression predicts a poor prognosis in HCC patients in the ZZU cohort. (a) Kaplan-Meier survival curves showing the correlation between UGP2 expression levels and the OS of HCC patients in the ZZU cohort. (b) Kaplan-Meier analysis revealed that OS was short in HCC patients in the ZZU cohort with low UGP2 expression levels regardless of the TNM stage. UGP2: uridine diphosphate-glucose pyrophosphorylase 2; HCC: hepatocellular carcinoma; OS: overall survival; TNM: tumour-node-metastasis.

To assess the prognostic value of UGP2 in HCC, Kaplan-Meier analysis, and a log-rank test were used to analyse the relationship between UGP2 expression and clinical follow-up information. The results showed that patients with lower UGP2 mRNA expression levels had significantly lower OS rates (*P* = 0.022; Figure 4(a)) and shorter progression-free survival (PFS) times (*P* = 0.011; Figure 4(b)) than those with higher UGP2 expression levels in the TCGA dataset. Furthermore, the association between UGP2 expression and OS/PFS in early- and advanced-stage HCC was analysed by the Kaplan-Meier method. The results indicated that the OS/PFS of HCC patients with lower UGP2 expression was shorter regardless of the tumour-node-metastasis (TNM) stage (*P* < 0.0001; Figures 4(c) and 4(d)**)**. In addition, GSEA also revealed that gene sets negatively correlated with the survival of HCC patients were enriched in the group with low UGP2 expression levels (*P* < 0.001; Figure 4(e)), while gene sets positively correlated with the survival of HCC patients were enriched in the group with high UGP2 expression levels (*P* < 0.001; Figure 4(f)). The prognostic value of UGP2 at the protein level was also assessed in the ZZU HCC cohort. Consistent with the aforementioned results, patients with lower UGP2 expression levels had a shorter survival time than those with higher UGP2 expression levels (*P* = 0.003; Figure 5(a)), and the OS of HCC patients with lower UGP2 expression levels was shorter regardless of the TNM stage (TNM I/II: *P* = 0.048; TNM III/IV: *P* = 0.043; Figure 5(b)).

To further confirm the clinical significance of UGP2 expression in HCC, the ZZU HCC cohort was used. The clinicopathological characteristics of the HCC patients are summarized in Table 1. Downregulated UGP2 expression was correlated with TNM stage (*P* = 0.014). There was no significant correlation between UGP2 expression and patient sex, age, liver cirrhosis status, alpha-fetoprotein (AFP) level, tumour multiplicity, tumour size, or portal vein thrombosis status (Table 1). Moreover, univariate and multivariate Cox regression analyses were used to determine the risk factors associated with patient prognosis in the ZZU HCC cohort. The univariate analysis showed that UGP2 expression, TNM stage, and portal vein thrombosis were significant prognostic factors for OS (Table 2). Furthermore, the multivariate analysis demonstrated that UGP2 expression and TNM stage were independent predictors of OS in HCC patients (Table 2). These data suggested that low UGP2 expression may be a predictor of a poor prognosis in HCC patients.

### 3.5. Potential Molecular Mechanism of the UGP2-Mediated Progression of HCC

**Figure 6:**
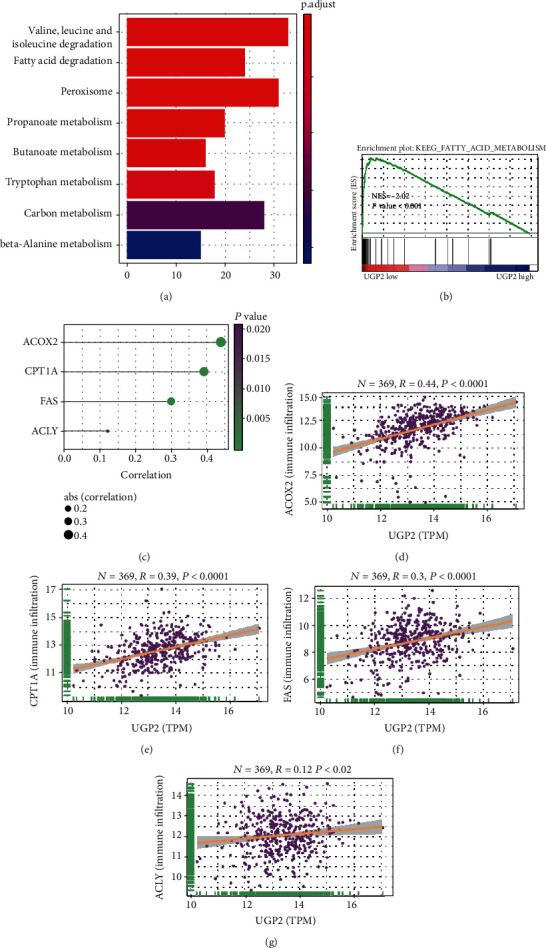
UGP2 has potential roles in fatty acid metabolism. (a) KEGG functional enrichment analysis showing the potential mechanism of UGP2 in HCC. (b) GSEA of the relationship between low UGP2 expression and genes associated with fatty acid metabolism. (c–g) The scatter plots show that UGP2 expression is markedly correlated with genes involved in fatty acid metabolism. UGP2: uridine diphosphate-glucose pyrophosphorylase 2; KEGG: Kyoto Encyclopedia of Genes and Genomes; HCC: hepatocellular carcinoma.

To detect the potential molecular mechanism of UGP2 in HCC, KEGG functional enrichment analysis and GSEA were used. A significant correlation between low UGP2 expression and fatty acid metabolism was identified (Figures 6(a) and 6(b)). To further confirm the functional roles of UGP2 in fatty acid metabolism, scatter plot analysis was used to assess the association between UGP2 and the genes involved in the fatty acid metabolism pathway. As presented in Figures 6(c)–6(g), UGP2 was significantly correlated with ACOX2 (*P* < 0.0001; *r* = 0.44; Figure 6(d)), CTP1A (*P* < 0.0001; *r* = 0.39; Figure 6(e)), FAS (*P* < 0.0001; *r* = 0.3; Figure 6(f)), and ACLY (*P* = 0.02; *r* = 0.12; Figure 6(g)). Taken together, these observations indicated that UGP2 may play essential roles in fatty acid metabolism.

## 4. Discussion

Although advances in HCC therapy have been achieved in recent decades, HCC remains one of the diseases with the worst prognosis among all malignant tumour types [19–21]. Following HCC diagnosis, most patients are in the middle and late TNM stages [22–24]. Thus, it is particularly important to discover novel biomarkers for improving HCC prognosis.

Previous studies have reported abnormal expression levels of UGP2 in various cancer types, including pancreatic ductal carcinoma [12], gallbladder cancer [13], colorectal cancer [14], acute myeloid leukaemia [15], glioma [16], and HCC [17]. Based on TCGA datasets, GEO datasets, the pancancer TMA, and the ZZU HCC cohort data, the current study demonstrated that UGP2 expression was downregulated at both the mRNA and protein levels in HCC tissues.

Although a previous study reported that UGP2 exhibits the potential to differentiate MR from NR HCC patients [17], there is no convincing evidence of its diagnostic value in HCC. Therefore, the present study conducted ROC curve analyses of UGP2 data obtained from the TCGA and GEO datasets. The AUC values of the ROC curve analysis for UGP2 were 0.7725, 0.7050, 0.9352, 0.8129, 0.7787, and 0.8826, which showed good diagnostic performance. The general criteria used for the AUC are as follows: 0.5-0.7 indicates that the effect is low but valuable, 0.7-0.85 indicates a general effect, 0.85-0.95 indicates a good effect, and 0.95-1 indicates a very good but unlikely effect [25]. These results suggested that UGP2 showed good diagnostic potential for differentiating HCC from non-HCC tissues.

Previous studies have correlated low UGP2 expression in many tumour types with tumour progression and poor prognosis. For example, Wang et al. [12] reported that the expression level of UGP2 is closely associated with the tumourigenesis and progression of pancreatic malignancy and that it can serve as a valuable prognostic factor in pancreatic cancer. Similarly, UGP2 is also a potential biomarker for the progression and prognosis of gallbladder cancer [13] and human glioma [16]. The present study revealed that the UGP2 expression level was positively correlated with the prognosis of HCC patients in the TCGA database and those in the ZZU HCC cohort. In addition, GSEA demonstrated that a lower UGP2 expression level was correlated with gene signatures associated with poor survival, while a higher UGP2 expression level was correlated with gene signatures associated with good survival. Furthermore, univariate and multivariate analyses confirmed that UGP2 expression was an independent factor for a poor prognosis in HCC patients.

Systematic bioinformatics analysis was also performed to predict the potential mechanism of UGP2 in HCC, which demonstrated a significant correlation between low UGP2 expression and fatty acid metabolism. A number of studies have noted a relationship between abnormal fatty acid metabolism and the occurrence and development of several tumours [26–29], including HCC [30–32]. We found that the UGP2 expression level was closely correlated with ACOX2, CTP1A, FAS, and ACLY, which are involved in fatty acid metabolism. These results suggested that UGP2 may play an important role in the occurrence and development of HCC by regulating fatty acid metabolism. However, further studies are needed to confirm this hypothesis.

## 5. Conclusion

In summary, the present study demonstrated that UGP2 expression is significantly downregulated in HCC tissues. A low expression level of UGP2 is positively correlated with a poor prognosis in HCC patients and can specifically distinguish between HCC tissues and matched adjacent nontumour tissues. These results suggest that UGP2 may serves as a novel prototype therapeutic agent for HCC patients.

## Figures and Tables

**Table 1:** Correlation of clinicopathological characteristics with UGP2 expression in the ZZU HCC cohort.

Variable	All patients (%)	Low UGP2	High UGP2	*P* value
Total	335	192	143	
Sex				0.276
Female	73 (21.8)	46	27	
Male	262 (78.2)	146	116	
Age (years)				0.317
<50	125 (37.3)	74	51	
≥50	210 (62.7)	118	102	
Liver cirrhosis				0.386
Yes	309 (92.2)	175	134	
No	26 (7.8)	17	9	
AFP (ng/ml)				0.529
<20	162 (48.4)	90	72	
≥20	173 (51.6)	102	71	
Tumour multiplicity				0.969
Single	255 (76.1)	146	109	
Multiple	80 (23.9)	46	34	
Tumour size (cm)				0.563
≤5	186 (55.5)	104	82	
>5	149 (44.5)	88	61	
Portal vein thrombosis				0.195
Absence	296 (88.4)	165	131	
Gross	39 (11.6)	26	13	
TNM stage				0.014^∗^
Early stage (I-II)	262 (78.2)	141	121	
Late stage (III-IV)	73 (21.8)	51	22	

Notes: ^∗^*P* < 0.05, ^∗∗^*P* < 0.001. Abbreviations: AFP: alpha-fetoprotein; TNM: tumour-node-metastasis.

**Table 2:** Univariate and multivariate Cox regression analyses of risk factors for overall survival time in the ZZU HCC cohort.

Variable	Univariate analysis		Multivariate analysis	
	Hazard ratio (95% CI)	*P* value	Hazard ratio (95% CI)	*P* value
Sex (male vs. female)	1.145 (0.715-1.831)	0.573		
Age (<50 vs. ≥50 years)	0.968 (0.651-1.440)	0.874		
Liver cirrhosis (absent vs. present)	1.557 (0.834-2.907)	0.165		
AFP (≥20 ng/ml vs. <20 ng/ml)	0.760 (0.516-1.120)	0.165		
Tumour multiplicity (single vs. multiple)	1.023 (0.653-1.604)	0.919		
Maximal tumour size (<5 cm vs. ≥5 cm)	1.019 (0.694-1.497)	0.923		
Portal vein thrombosis (absence vs. gross)	0.543 (0.327-0.903)	0.019^∗^	0.987 (0.566-1.721)	0.963
TNM stage (I-II vs. III-IV)	0.317 (0.213-0.472)	0.000^∗∗^	0.332 (0.215-0.513)	0.000^∗∗^
UGP2 level (low vs. high)	1.873 (1.245-2.819)	0.003^∗^	1.754 (1.162-2.648)	0.007^∗^

Notes: ^∗^*P* < 0.05, ^∗∗^*P* < 0.001. Abbreviations: AFP: alpha-fetoprotein; TNM: tumour-node-metastasis; HR: hazard ratio; CI: confidential interval.

## Data Availability

The datasets used and/or analysed during the current study are available from the corresponding author on reasonable request.
